# Topical over Dermal Versus Transdermal Application of Cyanoacrylate in Wound Synthesis and Its Effects on Healing—Experimental Study

**DOI:** 10.3390/bioengineering12111147

**Published:** 2025-10-23

**Authors:** Inácio Silva Viana, Paula Alessandra Di Filippo, Gabriel João Unger Carra, Francielli Pereira Gobbi, Lara Souza Ribeiro, Rachel Bittencourt Ribeiro, Fernando Antônio M. Petri, Maria Luíza Favero, Luíza Maria Feitosa Ribeiro, Eulogio Carvalho Queiroz Carvalho, Paulo Aléscio Canola

**Affiliations:** 1 Department of Veterinary Clinics and Surgery, Faculty of Agricultural and Veterinary Sciences, São Paulo State University (Unesp), Jaboticabal 14884-900, São Paulo, Brazil; gabriel.carra@unesp.br (G.J.U.C.); fernando.petri@unesp.br (F.A.M.P.); maria.favero@unesp.br (M.L.F.); 2 Laboratory of Animal Clinics and Surgery, Center for Agricultural Sciences and Technologies, State University of Northern Fluminense “Darcy Ribeiro”, Campos dos Goytacazes 28013-602, Rio de Janeiro, Brazil; pdf@uenf.br (P.A.D.F.); franci_gobbi@hotmail.com (F.P.G.); f.feitosaribeiro@gmail.com (L.M.F.R.); 3 Laboratory of Animal Pathology and Morphology, Center for Agricultural Sciences and Technologies, State University of Northern Fluminense “Darcy Ribeiro”, Campos dos Goytacazes 28013-602, Rio de Janeiro, Brazil; lara.ribeiro@uenf.br (L.S.R.); rachel_bittencourt@hotmail.com (R.B.R.); eulogio@uenf.br (E.C.Q.C.)

**Keywords:** topical adhesive, super glue, wound, inflammation, cytotoxicity, surgery

## Abstract

Cyanoacrylate-based adhesives are commonly used for wound closure due to their short synthesis time, aesthetic outcomes, and minimal discomfort. However, reported adverse effects include the release of cytotoxic metabolites, inflammation, and foreign body reactions. This study evaluated and compared the effectiveness of three cyanoacrylate-based adhesives for skin incision closure in *Rattus norvegicus*. The subjects were divided into three groups based on the type of monomer: G1 (n-2-ethyl-cyanoacrylate), G2 (n-2-butyl-cyanoacrylate), and G3 (n-2-octyl-cyanoacrylate). Each animal received two 2 cm paramedian incisions, which were closed using either a topical over dermal (OD) or a topical transdermal (TD) application, resulting in two subgroups per group. Wounds were evaluated on postoperative days 3, 7, 14, and 21 to compare the different monomers and application techniques. Assessment of the inflammatory infiltrate revealed differences in polynuclear cells between the TD and OD on days 3 and 7, while TD demonstrated improved results in mononuclear cells at all time points. Sustained inflammatory processes and foreign body reactions were observed. Quantification of tumor necrosis factor (TNF-α) and thiobarbituric acid reactive substances (TBARS) indicated that TD maintained stability throughout the assessment periods, though it exhibited higher values than OD from days 7 to 21. These higher values were associated with a foreign body reaction and increased oxidative stress. Regarding tissue formation, OD produced more aligned wound edges, supporting the production of types I and III collagen and improving scar resolution compared to TD. Our findings indicate that the patch application technique has a greater impact on healing than the size of the cyanoacrylate monomer.

## 1. Introduction

Sutures are the most common routine surgical procedure, used in both trauma and surgical treatments [1]. Although they are recognized as the gold standard for wound closure, intensive suturing can be tiring and time-consuming, and it can sometimes harm the patient. Sutures increase surgical and anesthetic time and favor surgical bed contamination, post-surgical infections, secondary tissue damage, and poor aesthetic results [2]. For these reasons, alternative materials, such as staplers, sealants, and adhesives, both organic and inorganic, are used for wound closure [3].

Adhesives are the most widely used of these materials as an alternative to conventional sutures. They eliminate the need for an injection of local anesthetic, reduce the risk of needlestick injuries, and eliminate the need for suture removal. In this field, cyanoacrylate (CA)-based adhesives have stood out, offering easy application, rapid adhesive polarization, and Elimination of trauma at the periphery of the wound [4]. They also act as sealants and offer a good cost–benefit ratio. However, they are only indicated for use in high-tension tissue regions and mucous membranes [4,5]. Furthermore, cytotoxicity and an exacerbated exothermic effect have been observed. Several changes have been made to the polymer composition, including the length and complexity of its carbon chains, to generate molecules that include CAs methyl (R=CH3), ethyl (R=C2H5), butyl and isobutyl (R=C4H9), octyl (R=C8H17), and decyl-CA (R=C10H21). These changes aim to minimize the deleterious effects, which are a constant target of investigation [5].

In experimental and case studies on the topical application of CA adhesives in wound healing published over time, there is no consensus regarding the effects of CA adhesives [6,7,8,9]. Toxic degradation products resulting from the carbon chain length of the CA molecule, foreign body reactions, and dehiscence are still occasionally reported [7]. Furthermore, despite the indication for the topical application of CA adhesives, two topical application techniques were noted [10,11]. The first technique involves approximating the wound edges, followed by topical application [7,8,9]. The second technique involves topical application of the CA adhesive to the wound edges, followed by approximation [10,11,12].

Based on these conjectures, we hypothesize that the size of the CA molecule and its application method may directly impact the inflammatory process, thereby altering the healing process and, consequently, the clinical application of these polymers. Thus, the present study aimed to evaluate inflammation and the characteristics of the healing process by comparing three different CA monomers (ethyl, butyl, and octyl-CA), with topical application to dermal (OD) and transdermal (TD) skin for the closure of experimental surgical wounds in *Rattus norvegicus*.

## 2. Material and Methods

This study was approved by the Ethics and Welfare Committee of the State University of Northern Rio de Janeiro under protocol 328/2017.

### 2.1. Animal Experimentation

Eighty-four male, non-castrated Wistar rats (*Rattus norvegicus*), over 10 weeks old and free of disease, were evaluated. The animals were housed individually under standardized environmental conditions (23 ± 1 °C with 55 ± 5% humidity and a 12-h light/dark cycle) and had free access to drinking water via a stainless-steel drinking fountain as well as specific rodent chow (Presence-Cravinhos, SP, Brazil).

### 2.2. Wound Creation

To create the skin wounds, the animals were anesthetized with a combination of 10% ketamine hydrochloride (Syntec—Santana de Parnaíba, SP, Brazil) at 100–200 mg/kg and 2% xylazine hydrochloride (Syntec—Santana de Parnaíba, SP, Brazil) at 5–16 mg/kg, which was administered intraperitoneally [13].

After anesthesia was administered, the ventral thoracoabdominal region was shaved and cleaned with a 2% chlorhexidine gluconate solution (Dermo Suave— Rio de Janeiro, RJ, BR) and 70% ethyl alcohol solution (Exodo—Sumaré, SP, Brazil). Then, two 2-cm-long paramedian skin incisions were made on both sides of the linea alba.

### 2.3. Experimental Design

After wound creation, the animals were randomly assigned to three groups based on the carbon chain length of each CA adhesive. Each group consisted of 28 animals:

- Ethyl-cyanoacrylate (Three Bonder^®^—São Paulo, SP, Brazil);
- Butyl-cyanoacrylate (Histoacryl^®^, B. Braun—Melsungen, Germany);
- Octyl-cyanoacrylate (Liqui Band^®^—Winsford, UK).

Each group was then divided into two subgroups according to the wound closure technique:

- Topical over dermal (OD); approximation of the wound edges with anatomical forceps, followed by application of CA (right abdominal wound);
- Topical transdermal (TD); application to the wound edges, followed by approximation of the edges with anatomical forceps (left abdominal wound).

For analgesia, 16 mg/kg, SC, BID, 3 days of dipyrone (Dorfin^®^—Saint-Laurent, QC, Canada) was administered. In the postoperative period, no hygiene or application of any other substances was performed.

### 2.4. Sample Collection and Analysis

On postoperative days 3, 7, 14, and 21, seven animals from each group were euthanized by administering pentobarbital (Pisabental^®^, São Paulo, SP, Brazil) 100 to 200 mg/kg intraperitoneally [14], followed by necropsy and collection of samples for macroscopic and histopathological analyses. The macroscopic parameters evaluated were the presence of polymer, dehiscence, necrosis, the presence of seroma, and the presence of exudate. For histological and immunohistochemical evaluations, skin samples from the wound area, including the dermis, epidermis, and subcutaneous tissue, were fixed in 10% buffered formalin. For biochemical analyses, the samples were stored at −80 °C until testing.

### 2.5. Histomorphometry

Histomorphometric evaluation was performed after routine histological processing with 5 μm sections arranged on clean, dry histological slides, and stained with hematoxylin and eosin (H/E) and Picrosirius special red. Two slides of each sample were prepared and analyzed using light and polarized light optical microscopy for both staining procedures. Slide images were obtained using an Olympus BX53-P microscope (Olympus Life Science, Tokyo, Japan) and cellSens Standard software version 4.3.1 for image capture, with magnification of 40× (two images per slide) and 400× (four images per slide). Subsequently, the images were evaluated and quantified for the presence of poly- and mononuclear infiltrates, fibroblasts, blood vessels, and types I and III collagen using the “Cell Counter,” “Threshold,” and “IHC Toolbox” plugins of Fiji ImageJ software (NIH Image Software, Vancouver, BC, Canada).

### 2.6. Immunohistochemistry

The samples were sliced at 5.0 μm thickness, placed on previously silanized (3-aminopropyltriethoxysilane) slides (Sigma-Aldrich, Saint Louis, MO, USA), and subjected to antigen retrieval in citrate buffer (pH 6.0). The samples were incubated overnight with primary antibodies: anti-VEGF at a 1:400 dilution (Cat. ab1316, Abcam, Cambridge, UK), anti-TNF-α at a 1:200 dilution (Cat. ab1793, Abcam, Cambridge, UK), and anti-TGF-β1 at a 1:100 dilution (Cat. NBP2-45137, Novusbio, Centennial, CO, USA). They were detected using a Novolink™ Max Polymer Detection System (Leica Biosystems, Newcastle, UK) according to the manufacturer’s instructions and counterstained with hematoxylin [15].

Four fields from each slide were obtained and quantified at 400× magnification using the “IHC Toolbox” plugin of the ImageJ software to determine the percentage of positive areas in the captured images for TNF-α, TGF-β1, and VEGF. Results were expressed as a percentage of positively stained cells.

### 2.7. Oxidative Stress

Oxidative stress was quantified through lipid peroxidation by quantifying thiobarbituric acid-reactive substances (TBARS). The sample (100 mg) was homogenized with the aid of Ultra-Turrax^®^ (T10, IKA, Campinas, SP, Brazil) in 1.0 mL of 1.15% KCl. Then, 3.0 mL of pure water and 0.5 mL of 2 M NaOH were added and heated at 60 °C for 30 min in a water bath and neutralized at the end with 1.0 mL of 2 M HCl. Subsequently, 1 mL of 2 M thiobarbituric acid was added and heated in a water bath at 100 °C for 30 min. This reaction produces a yellow compound that is measured using a spectrophotometer at 540 nm. The results are expressed as TBARS/OD/mg of tissue [16].

### 2.8. Hydroxyproline (HO-Pro)

Skin fragments collected from the lesion area were used to quantify hydroxyproline. The samples were dehydrated in acetone for 48 h (changed every 24 h) and chloroform-ethanol (2:1) solution for 48 h (changed every 24 h). After dehydration, the fragments were dried at 60 °C for 2 h and hydrolyzed in 6 M HCl (1 mL/10 mg of tissue) at 130 °C for 2 h. Subsequently, 20 μL of the hydrolyzed sample, 50 μL of chloramine T solution and 50 μL of Ehrlich reagent were placed in 96-well plates in duplicate, followed by incubation at 65 °C for 15 min. The absorbance of the samples was read at 550 nm, using a standard curve ranging from 0.2 to 6.0 μg of trans-4-hydroxy-L-proline (Cat. H54409, Sigma-Aldrich, Saint Louis, MO, USA) [17].

### 2.9. Glycosaminoglycans (GAG)

The 1,9-dimethylmethylene blue (DMMB) test was used to quantify sulfated glycosaminoglycans. The sample was dehydrated in acetone for 24 h and dried at 37 °C for 2 h. Subsequently, the sample was weighed and digested with papain (40 mg/1 g of tissue) at 50 °C for 24 h. After digestion, papain was precipitated with ethanol for 12 h, dried at 37 °C for 2 h and resuspended in 35 μL of water. In duplicate, 10 μL of the sample and 250 μL of DMMB solution were added to the 96-well plate for quantification of GAGs. The absorbance was measured at 526 nm, using a standard curve ranging from 0.31 to 5.0 μg of sodium chondroitin sulfate (Cat. Y0000280, Sigma-Aldrich, Saint Louis, MO, USA) [18].

### 2.10. Statistical Analysis

All data were expressed as mean ± SD. Student’s *t*-test was used to compare the forms of CA application within the same group and at the same time point. One-way analysis of variance (ANOVA), followed by a Tukey post hoc test, it was used to compare the different groups and evaluation times. The nonparametric Wilcoxon paired test was used to compare values obtained from the two application forms (OD and TD) within a group at the same evaluation period. GraphPad Prism 8.0.1 software was used to perform the statistical analyses and construct the graphs, setting the significance level at *p* < 0.05.

## 3. Results

### 3.1. Inflammatory Infiltrate

**Figure 1:**
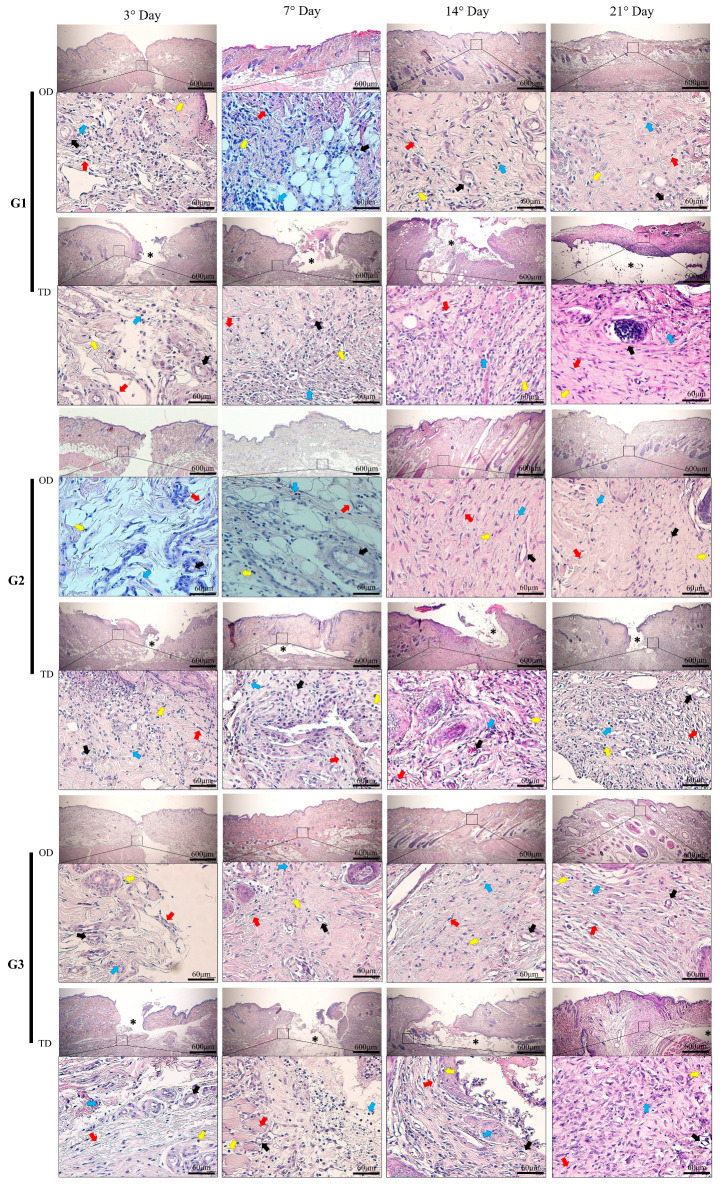
Histopathological photomicrographs of the CA-based adhesive application area (*) at 40× magnification and identification of scar elements at 400× magnification. On postoperative days 3 and 7, areas of tissue edema and disorganized connective tissue formation and areas of granulation tissue are shown, along with the presence of polynuclear cells (blue arrows), mononuclear cells (yellow arrows), and blood vessels (black arrows). On days 14 and 21, tissue reorganization can be observed in wounds treated with OD application and formation of fibrous connective tissue around the adhesives with a large number of fibroblasts (red arrows) in wounds treated with TD application. CA groups: n-2-ethyl-cyanoacrylate (G1), n-2-butyl-cyanoacrylate (G2), and n-2-octyl-cyanoacrylate (G3).

Histomorphological analysis to quantify the inflammatory index indicated that neutrophils and eosinophils predominated among polymorphonuclear cells and macrophages among mononuclear cells (Figure 1).

**Figure 2:**
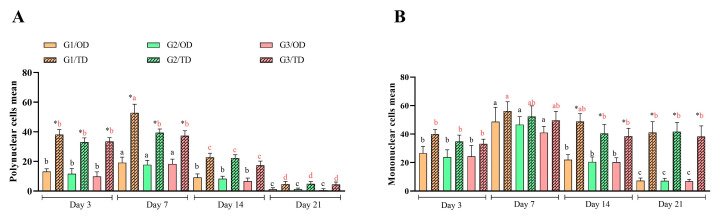
(**A**): indicates a polynuclear infiltrate. (**B**): indicates a mononuclear infiltrate. CA groups: n-2-ethyl-cyanoacrylate (G1), n-2-butyl-cyanoacrylate (G2), and n-2-octyl-cyanoacrylate (G3). Bar charts use black letters for over dermal (OD) and red letters for transdermal (TD) applications. Within each assessment, matching colored letters in the corresponding columns indicates no significant differences between the groups during the evaluation period (Tukey’s post hoc test; *p* < 0.05). (*) marks statistical differences related to the site of CA application (Wilcoxon test; *p* < 0.05).

The peak of polymorphonuclear infiltration was observed on postoperative day 7, followed by a reduction at later time points in the OD group (*p* < 0.05) (Figure 2A). With TD application of the patch, the polymorphonuclear inflammatory index levels were maintained from day 3 to 21, with the exception of ethyl-CA (G1) on day 7, which showed superior results (*p* < 0.05). Significant differences between the OD and TD application methods were evident in all groups from day 3 to 7 (*p* < 0.05).

As with the polymorphonuclear infiltrate, the mononuclear infiltrate increased from day 3 to day 7, followed by reductions on days 14 and 21 in the OD application of the adhesive (*p* < 0.05) (Figure 2B). Unlike the OD application, the TD application, the number of mononuclear cells remained high at all evaluation time points, especially in the area of contact between the tissue and the adhesive, with emphasis on ethyl-CA (G1) on day 7 compared to the other evaluation time points (*p* < 0.05).

In the evaluation between the OD and TD application techniques within the groups, the TD application showed superior results on days 14 and 21 (*p* < 0.05). There were no significant differences between the types of CA adhesives when comparing the monomers under the same adhesive application technique and the same evaluation time point.

### 3.2. Inflammation and Oxidative Stress

**Figure 3:**
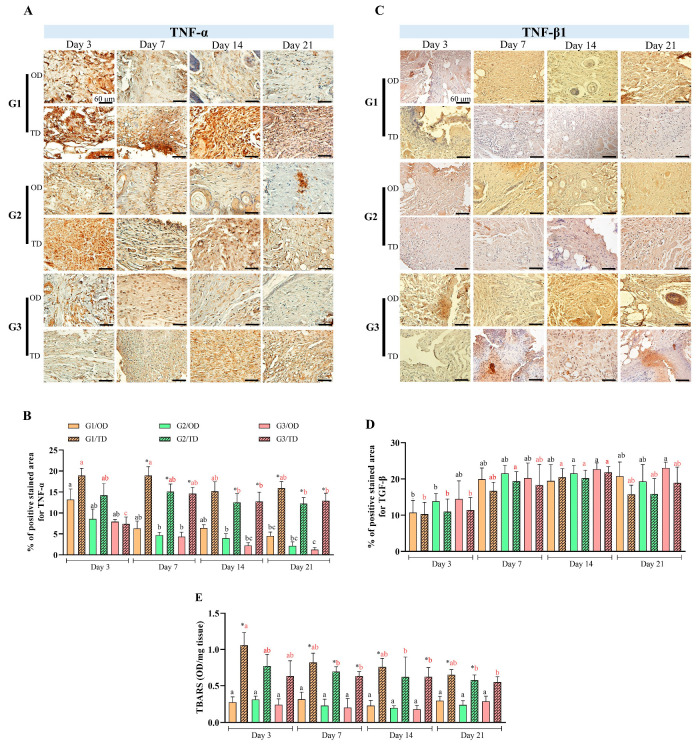
(**A**): TNF-α immunohistochemical images at 400× magnification. (**B**): Quantified TNF-α positive areas (%). (**C**): TGF-β1 immunohistochemical images at 400× magnification. (**D**): Quantified TGF-β1 positive areas (%). (**E**): TBARS assay for oxidative stress measurement. CA groups: n-2-ethyl-cyanoacrylate (G1), n-2-butyl-cyanoacrylate (G2), and n-2-octyl-cyanoacrylate (G3). Black letters on the bars refer to OD, and red letters refer to TD. Identical letters within a color across different columns indicate no significant difference (Tukey’s test; *p* < 0.05). (*) marks statistical differences related to the site of CA application (Wilcoxon test; *p* < 0.05).

The immunohistochemical evaluation revealed that TNF-α exhibited a larger positive area on the third postoperative day. Statistically significant differences between OD and TD were observed from the seventh to the twenty-first day (*p* < 0.05) (Figure 3A,B). On the 21st postoperative day, TNF-α expression was higher in the ethyl-CA group (G1) than in the octyl-CA group (G3) in the OD groups (*p* < 0.05). In the TD group, octyl-CA (G3) demonstrated distinct characteristics on the third postoperative day compared to the other groups and time points. TNF-α expression levels were higher on postoperative days 3 and 7 for the ethyl-CA group (G1), and on days 14 and 21 for the butyl (G2) and octyl-CA (G3) groups.

Significant differences in TGF-β1 levels were primarily observed on the third and fourteenth postoperative days (*p* < 0.05), regardless of the type of monomer or method of adhesive application used (Figure 3C,D). The TD groups exhibited a higher number of inflammatory cells than the OD group. TD was associated with higher neutrophil and M1 macrophage activity, resulting in increased TNF-α production. In contrast, OD exhibited greater M2 macrophage activity, which is involved in TGF-β1 release, despite having lower mononuclear cell counts than the TD group.

Although the TD groups have a higher number of inflammatory cells than the OD, the correlation between inflammatory markers demonstrates that greater activity of neutrophils and M1 macrophages in the production of TNF-α was observed in the TD. Likewise, there was greater activity of M2 macrophages in releasing TGF-β1 in the OD group, despite having a lower number of mononuclear cells than the TD group.

Significant differences in oxidative stress (*p* < 0.05) were found between OD and TD for ethyl-CA (G1) at every time point, for butyl-CA (G2) on days 7 and 21 postoperative, and for octyl-CA (G3) on days 7 and 14 (Figure 3E). No notable distinctions were identified among monomers regarding OD during the assessment periods. On the third postoperative day in TD, ethyl-CA (G1) showed higher oxidative stress than octyl-CA (G3) (*p* < 0.05).

### 3.3. Neovascularization

**Figure 4:**
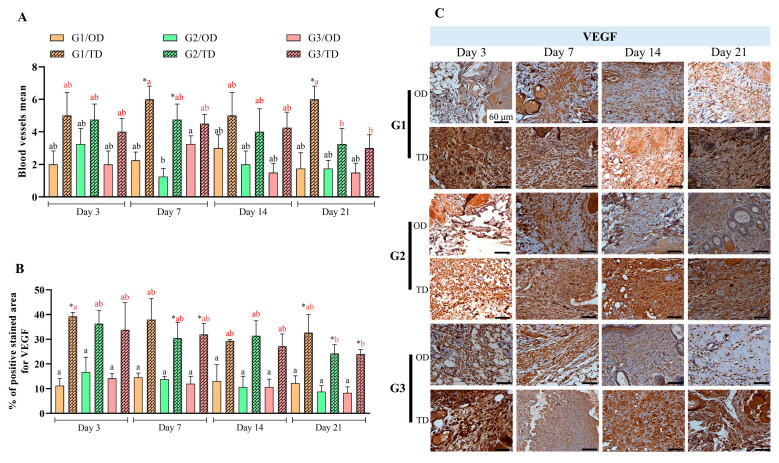
(**A**): Bood vessels quantification by histomorphometry. (**B**): VEGF positive areas (%). (**C**): VEGF immunohistochemistry images at 400× magnification (scale bar: 40 μm). CA groups: n-2-ethyl-cyanoacrylate (G1), n-2-butyl-cyanoacrylate (G2), and n-2-octyl-cyanoacrylate (G3), as well as evaluation times; black letters on the bars for OD and red letters on the bars for TD, in both evaluations the same letters with the same colors in different columns did not differ from each other (Tukey’s test; *p* < 0.05). (*) marks statistical differences related to the site of CA application (Wilcoxon test; *p* < 0.05).

Significant differences in blood vessel neovascularization were found between the application methods (OD and TD) of ethyl-CA (G1) on postoperative days 7 and 21, as well as for butyl-CA (G2) on postoperative day 7 (*p* < 0.05) (Figure 4A). In the OD group, butyl-CA was associated with a more pronounced neovascularization process, while octyl-CA showed the lowest rate of new blood vessel formation on postoperative day 7 (*p* < 0.05). In the TD group, neovascularization in ethyl-CA (G1) on postoperative day 21 differed from that of butyl-CA (G2) and octyl-CA (G3) (*p* < 0.05).

Analysis of VEGF-positive regions (Figure 4B,C) demonstrated statistically significant differences between the application methods (OD and TD) for ethyl-CA (G1) on day 3 (*p* < 0.05). Significant differences between TD and DO were also observed for butyl-CA (G2) and octyl-CA (G3) on day 7 (*p* < 0.05), as well as among all monomers on day 21 postoperatively (*p* < 0.05). No statistical differences were observed in the OD group between the periods. In TD, only ethyl-CA (G1) on day 3 differed from butyl-CA and octyl-CA (G2 and G3) on day 21 (*p* < 0.05).

### 3.4. Tissue Formation

**Figure 5:**
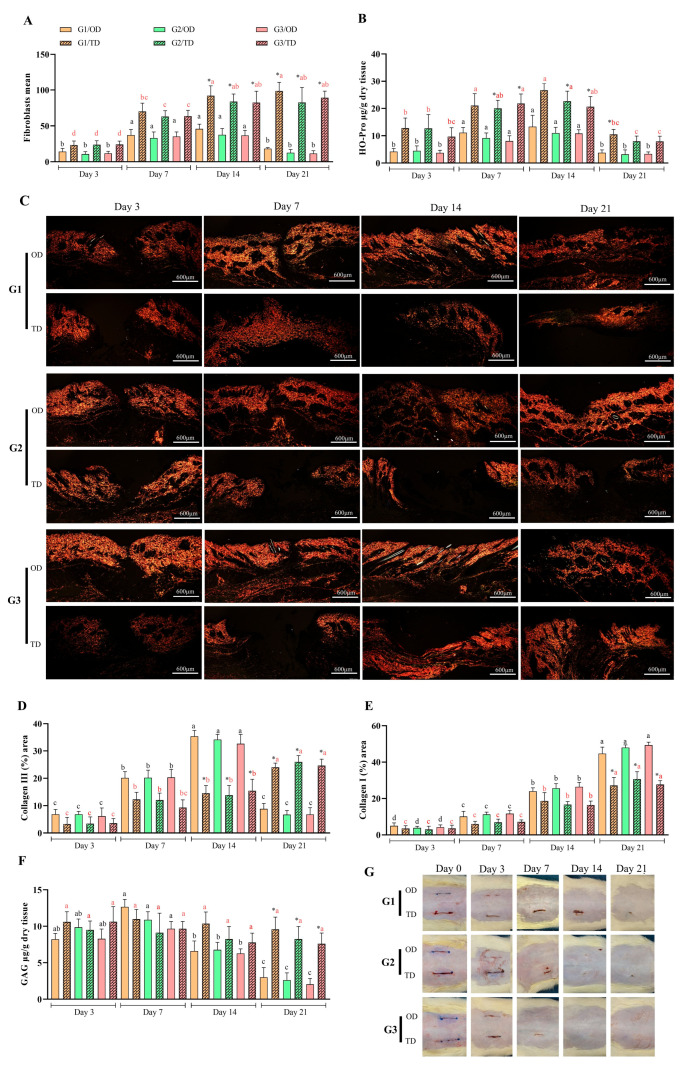
(**A**): Fibroblasts quantified using histomorphometry. (**B**): Hydroxyproline measured through tissue biochemistry. (**C**): Photomicrographs of picrosirius red staining at 40× magnification (scale bar: 600 μm), showing blackened areas of interruption between the healthy tissue and the lesion area, with reduction over the evaluated time points, especially in the OD, compared to the TD. (**D**,**E**): Collagen types III and I quantified as percentages, respectively. (**F**): Glycosaminoglycans (GAGs) assessed using tissue biochemistry. (**G**): Images depicting the use of cyanoacrylate (CA)-based adhesives and distinct stages of surgical wound healing. CA groups: n-2-ethyl-cyanoacrylate (G1), n-2-butyl-cyanoacrylate (G2), and n-2-octyl-cyanoacrylate (G3), as well as evaluation times; black letters on the bars for OD and red letters on the bars for TD, in both evaluations the same letters with the same colors in different columns did not differ from each other (Tukey’s test; *p* < 0.05). (*) marks statistical differences related to the site of CA application (Wilcoxon test; *p* < 0.05).

Fibroblast levels differed between OD and TD from days 14 to 21 postoperative for all monomers (*p* < 0.05) (Figure 5A). Elevated fibroblast levels were detected in the OD group on postoperative days 7 and 14, without significant differences among the CA monomers during these intervals. In the TD group, the presence of fibroblasts was lower on the third postoperative day (*p* < 0.05), gradually increasing in later assessments and reaching its highest level by day 21. No significant differences were observed between monomers at any evaluation point. The migration behavior of fibroblasts in the TD group closely resembled that of macrophages, with both cell types exhibiting pronounced accumulation in proximity to the CA polymer (Figure 2).

Significant differences in hydroxyproline quantification were found between OD and TD application techniques: for butyl-CA (G2) and octyl-CA (G3) on day 7, for all monomers on day 14, and for ethyl-CA (G1) on day 21 (all *p* < 0.05). In OD, hydroxyproline quantification increased on postoperative days 3 and 7 (*p* < 0.05) and then decreased on postoperative days 14 and 21 (*p* < 0.05), with no significant differences observed between CAs over time. In TD applications, hydroxyproline levels increased from postoperative days 3 to 14 (*p* < 0.05), except for butyl-CA (G2) on postoperative day 7 and octyl-CA (G3) on postoperative day 14; after this period, a decrease was recorded (*p* < 0.05).

Significant differences in type III collagen levels were observed between OD and TD on postoperative days 14 and 21 (*p* < 0.05). Specifically, OD exhibited higher levels on day 14, whereas TD demonstrated increased levels on day 21 (*p* < 0.05) (Figure 5D). Type III collagen levels increased over time, peaking after OD application on postoperative day 14, before declining by postoperative day 21 (*p* < 0.05). In the TD group, measurements increased between postoperative days 3 and 7, as well as between postoperative days 14 and 21 (*p* < 0.05). There were no differences between CA monomers under the same form of application in the same period.

Both OD and TD showed sustained increases in type I collagen levels; notably, OD outperformed TD only at postoperative day 21 (*p* < 0.05) (Figure 5E). For OD, significant differences were observed at all time points (*p* < 0.05). In contrast, TD showed more gradual changes, with statistically significant differences detected at postoperative day 14 (*p* < 0.05). Variations in type I collagen were evident over time within groups but not between groups.

Differences in GAG quantification were found between OD and TD only on postoperative day 21. In OD, GAG levels decreased between postoperative days 14 and 21 (*p* < 0.05). TD showed stable values over time, with no significant changes (Figure 5F). No differences were found between groups or over time with any application method.

### 3.5. Macroscopic Evaluation

For the macroscopic assessment of various CA monomer application techniques, a rigid, semitransparent film was noted to adhere to the skin in the OD group, accompanied by effective approximation of the wound edges (Figure 5G). Film formation in the TD group was less visually evident because of the application method, though it could be identified by palpation. Both the OD and TD groups demonstrated effective wound edge approximation after application.

During the evaluation period, four wounds treated with CA TD presented dehiscence: one on day 3 treated with butyl-CA and two on day 7 treated with ethyl-CA. No dehiscence occurred in the wounds treated with CA upon application of OD.

Upon evaluation, the adhesives were found to be brittle. Between days 14 and 21, films were observed with greater prominence in OD syntheses using octyl-CA, followed by those using butyl-CA (Figure 5G). Following film removal, wounds treated with CA monomers using the OD technique exhibited complete wound closure by postoperative day seven. By day 21 after surgery, TD wounds treated with any CA monomer showed complete or partial healing (Figure 5G). In TD, the absence of CA was observed exclusively in cases of dehiscence, whereas its presence was confirmed in cases without dehiscence. Necrosis, exudate, and seroma were evaluated; however, these conditions were not detected in any of the wounds, regardless of the material or technique used for wound edge approximation.

## 4. Discussion

Cyanoacrylate adhesives are low-density liquid polymers that quickly polymerize to form a film on the applied surface when in contact with anionic substances. They are popular due to their ease of use and reduced incision synthesis time [19,20]. Despite their widespread use, studies have not reached a consensus on their application. This study evaluates various CA polymers and application techniques across different wound healing intervals, addressing cytotoxicity, healing rates, and medical applications.

During healing by primary intention, the inflammatory process typically occurs locally as part of tissue repair, beginning with neutrophil migration, followed by macrophages [21,22]. However, research on the use of CA for wound healing has yielded mixed findings regarding the degree of associated inflammation. This has resulted in uncertainty over its clinical and surgical utility. The present study identifies factors contributing to these differing reports. Wounds treated via OD application showed rapid healing, with changes mainly limited to evaluation times, decreased inflammation, and quick scar formation. In contrast, the TD application resulted in increased recruitment of inflammatory cells, variation in monomer poly- and morphonuclear cell infiltration, a foreign body response, and delayed healing, as shown in Figure 1 and Figure 2. The OD results align with those of previous studies [5,9,10,15,23], while the TD findings are consistent with those of studies in cervical and pancreatic surgery and abdominal mesh fixation in rabbits, in which lower absorption and increased cellular infiltration during healing were noted [12,19,24,25].

TNF-α, a pro-inflammatory protein, is produced at higher levels during the initial stage of wound healing, prompting the recruitment of immune cells [26]. Its levels decrease as healing progresses successfully [26], as observed in OD-treated wounds. However, for the TD application, TNF-α expression persisted across time points. Elevated macrophage recruitment and ongoing inflammation maintained the presence of M1 macrophages (with pro-inflammatory gene expression), leading to sustained TNF-α levels [27], as depicted in Figure 2A,B. While there were no significant differences between OD and TD, greater M2 macrophage activity was evident. These macrophages release TGF-β1, which supports improved healing.

In TBARS assays, no differences emerged between monomers or over time for the OD application method. When comparing adhesive application forms, TD outperformed OD at all measured times. These outcomes are attributed to a foreign body response involving high levels of pro-inflammatory cytokines, including IL-1β, IL-6, TNF-α, and IFNγ, which may induce Th1 differentiation via IL-12 secretion, thereby raising inducible nitric oxide synthase (iNOS) and reactive oxygen/nitrogen species (ROS/RNS) [28]. The exothermic reaction caused by molecular polarization and stabilizers also plays a role. Commercial adhesives reportedly exhibit a greater exothermic effect than surgical adhesives in structural analyses of ethyl-CA in body fluids [29]. Under acute ROS conditions due to tissue damage, the body releases antioxidants, such as glutathione (GSH), to neutralize ROS/RNS and mitigate oxidative stress [30]. Although antioxidant release was not quantified here, the differences between OD and TD suggest unequal compensatory antioxidant release due to variable inflammation and foreign body responses.

The cytotoxicity of CAs, mainly mediated by the inflammatory index, has already been reported in several in vitro [8,10,31,32] and in vivo [6,19,32,33] studies. However, other studies that also evaluated the application of CA for in vivo synthesis found no evidence of CA cytotoxicity, despite differences in the healing processes between the molecules [7,8,9,15,23,34]. Upon comparing the experimental designs, results, and images of the in vivo studies reporting cytotoxicity, it was found that all studies involved transdermal application of CA, regardless of the polymer’s carbon chain size [6,12,19,32,33]. According to Cerdá et al. [11], the smaller the CA molecule, the greater the exothermic effect of polarization, release, and accumulation of formaldehyde, which causes cell death and the release of oxygen free radicals, contributing to tissue loss, the release of several mediators, and local ischemia, necrosis, and tissue damage.

Based on the findings regarding inflammatory cells, TNF-α, and TBARS levels, this study suggests that the inflammatory index, as well as the dehiscence, ischemia, and necrosis described by previous authors [11], are more closely associated with the use of adhesive stabilizers and application methods that produce a foreign body response, as seen with TD, rather than intrinsic molecular cytotoxicity. Surgical closure with ethyl-CA molecules, such as those in Epiglu^®^, has not been reported to cause cytotoxicity in routine practice. However, the TD application has been linked to slower healing. OD application demonstrated faster healing, with significant improvements observed at selected time points, possibly due to limited adhesive-tissue interaction and adhesive loss to the environment.

Wound angiogenesis begins around three days post-injury via VEGF release, with vascular density peaking at fourteen days [35]. A rabbit trial using different CA monomers for hernia reduction and TD applications reported that vessel numbers peaked on day seven and then declined [19]. These results matched the patterns of OD applications, with larger VEGF-positive areas observed under TD, particularly on day 21. However, smaller areas were recorded for butyl- and octyl-CA. TD applications for rabbit tendon synthesis maintained VEGF values throughout the observed periods [36]. The differences in VEGF levels between groups reflect the healing process rather than any stimulatory effect attributable to isolated CA molecules.

Fibroblast presence typically rises during inflammation and falls as healing progresses [37,38]. OD application showed a peak in fibroblasts on postoperative days 7 and 14 followed by a decrease. TD resulted in a continuous increase in fibroblasts, which stabilized between days 14 and 21 forming a fibrotic capsule characteristic of a foreign body response [39]. The observed differences between OD and TD after day 14 indicate divergent healing processes.

Collagen fragmentation, which is mediated by gelatinases (MMP-2 and MMP-9) and collagenases (MMP-1 and MMP-8), increases hydroxyproline levels and stimulates extracellular matrix (ECM) formation [40]. Initially, the wound is filled with a loose type III collagen matrix, which is later replaced with type I collagen to provide strength and elasticity [21]. Hydroxyproline levels peaked on days 7 and 14, and the application method of the adhesive influenced the magnitude. Quantification of type III collagen was higher for OD application on days 7 and 14, and for TD application on days 14 and 21. Both methods showed peaks in type III collagen on day twenty-one, with notable differences between them. These data suggest that TD application results in greater gelatinase and collagenase activity, leading to delayed and lower collagen production. This finding corroborates earlier reports [9,10].

Glycosaminoglycans (GAGs) play a central role in the healing process by composing the ECM and managing cell growth and hydrostatic balance [41]. This study identified differences in GAG quantification only in OD between time points and in TD on the 21st day. For TD, GAG levels remained high throughout, likely due to persistent inflammation and disorganized ECM.

Macroscopic evaluation revealed dehiscence, but no infection was present, even in the absence of dressings. Several studies have considered the antimicrobial properties of CA adhesives to be a key benefit, with promising results against *Escherichia coli*, *Staphylococcus aureus*, *Staphylococcus epidermidis*, *Corynebacterium pseudodiphtheriticum*, *Klebsiella pneumoniae*, *Pseudomonas aeruginosa*, and *Staphylococcus aureus* [31].

According to Pascual et al. [6], polymers with longer carbon chains are more elastic and flexible, whereas shorter chains provide stronger adhesion but become rigid and fragile after polarization. Over time, these polymers appear brittle with reduced adhesion [7,8,9,10,11,42], as seen in OD applications; this was not observed in TD-treated wounds.

This study highlights relevant results regarding the healing process in wounds using CA; however, the size of the experimental wounds and the relatively small application area of the CA adhesives were limiting factors. Further evaluations are needed, especially in TD, regarding oxidative stress in prolonged post-application periods, resistance testing, and more detailed polarization mechanisms of CA-based adhesives.

## 5. Conclusions

In conclusion, our preclinical study confirmed that different topical application techniques for CA-based adhesives can interfere with the healing process during wound closure. Topical application of CA adhesives to the dermis (OD) demonstrated efficient approximation and juxtaposition of wound edges and reduced interaction between the adhesive and the tissue, resulting in rapid healing and the absence of foreign body reactions. No significant differences associated with the carbon chain length of the adhesives were observed using this application technique.

Unlike OD, topical transdermal (TD) application, while promoting approximation, presents poor juxtaposition of the edges, resulting in increased inflammation, foreign body reactions, delayed healing, and a higher risk of dehiscence. These deleterious effects were more prominent in wounds treated with CA adhesives with shorter carbon chains (ethyl-CA).

## Data Availability

The data generated and analyzed during this study are available from the corresponding author upon reasonable request.
